# Parthenolide induces proliferation inhibition and apoptosis of pancreatic cancer cells in vitro

**DOI:** 10.1186/1756-9966-29-108

**Published:** 2010-08-10

**Authors:** Jun-Wei Liu, Min-Xia Cai, Ying Xin, Qing-Song Wu, Jun Ma, Po Yang, Hai-Yang Xie, Dong-Sheng Huang

**Affiliations:** 1 Department of General Surgery, Sir Run Run Shaw Affiliated Hospital, Zhejiang University School of Medicine, Hangzhou 310016, PR China; 2 Key Lab of Combined Multi-Organ Transplantation, Ministry of Public Health and Department of Hepato-Biliary-Pancreatic Surgery, First Affiliated Hospital, Zhejiang University School of Medicine, Hangzhou 310003, PR China

## Abstract

**Background:**

To explore the anti-tumor effects of parthenolide in human pancreatic cancer.

**Methods:**

BxPC-3 cell, a human pancreatic cancer, was treated with parthenolide at different concentrations. The MTT assay was used to analyze cell viability. Flow cytometry and DNA fragmentation analysis were applied to evaluate apoptosis after parthenolide treatment. The wound closure and cell invasion assay were also employed in the study. Western blotting was used to demonstrate Bad, Bcl-2, Bax, caspase-9 and pro-caspase-3 expression.

**Results:**

The MTT assay indicated that the pancreatic cancer growth could be dose-dependently inhibited by parthenoolide. This phenomenon was confirmed by flow cytometry and DNA fragmentation analysis. The wound closure assay and cell invasion assay showed that BxPC-3 cell was significantly suppressed by parthenolide at 7.5 μM and 15 μM. Western Blotting demonstrated the Bcl-2 and pro-caspase-3 were down-regulated while the Bax and caspase-9 were up-regulated. No alteration in Bad expression was found after treatment.

**Conclusions:**

The parthenolide can inhibit the cell growth, migration, and induce the apoptosis in human pancreatic cancer. These findings may provide a novel approach for pancreatic cancer treatment.

## Background

Pancreatic adenocarcinoma is among the leading causes of cancer related mortality throughout the world [1]. Currently surgical resection is still the main therapeutic approach. However most cases are unresectable when diagnosed. Even in resectable cases, the long-term outcome remains unsatisfactory. The statistics disclosed that one-year survival rate was less than 10%, 5-year survival rate was less than 1% and median survival duration ranged from three to four months, respectively. The clinic reality mentioned above made chemotherapy essential for a cure. However drug-resistance can compromise the therapeutic effectiveness which is the major concern nowadays [2].

Parthenolide (PTL) is the main extracts of sesquiterpene lactone isolated from Mexican and Indian herbs such as feverfew (Tanacetum parthenium). PTL has been used conventionally to treat migraine and rheumatoid arthritis for centuries [3]. Recently it has been reported that PTL may induce inhibition of proliferation and apoptosis in various human cancer cells in vitro, such as colorectal cancer, hepatoma, cholangiocarcinoma [4-6]. In addition, PTL can sensitize resistant cancer cells to anti-tumor agents [7,8] and act as a chemo-preventive agent in an animal model of UVB-induced skin cancer [9]. Meanwhile data have showed that PTL-induced apoptosis is associated with inhibition of transcription factor nuclear factor-kappa B (NF-kB) [3,10], mitochondrial dysfunction and increase of reactive oxygen [11,12]. However the detailed molecular mechanisms of anticancer effect of PTL are largely unknown.

Our study disclosed that PTL induced apoptosis in BxPC-3 cells mainly by influencing bcl-2 family. PTL and its sesquiterpene lactone analogues might be new chemotherapeutic agents for pancreatic cancer.

## Methods

### Cell culture and reagents

Human pancreatic cancer cell line BxPC-3 was purchased from Shanghai Institute of Cell Biology, Chinese Academy of Sciences (Shanghai, China). It was cultured in dulbecco's modified eagle's medium (DMEM, HyClone, Logan, Utah, USA) containing 10% fetal bovine serum (JRH Biosciences, Lenexa, Kansas, USA), peniciline streptomycin mixture at 37°C in a humidified atmosphere of 5% CO2 and 95% air. Parthenolide (Sigma, St. Louis, MO, USA) supplied as a crystalline solid was dissolved in dimethylsulfoxide (100 mM stock) and stored at -20°C. Antibodies used in this study were obtained from Santa Cruz (CA, USA) and Cell Signaling Technology (CA, USA) respectively.

### MTT colorimetric survival assay

BxPC-3 cells were plated at a density of 1.0 × 10^4 ^cells per well in 96 well plates. 24 hours after incubation, cells were treated by PTL at indicated concentrations for 48 hours; then the medium was removed and 200 μl of fresh medium plus 20 μl of 3-(4, 5-dimethylthiazol-2yl)-2, 5-diphenyltetrazolium bromide (MTT, 2.5 mg dissolved in 50 μl of dimethylsulfoxide, Sigma, St. Louis, MO, USA) were added to each well. After incubation for 4 hours at 37°C, the culture medium containing MTT was withdrawn and 200 μl of dimethylsulfoxide(DMSO) was added, followed by shaking for 10 minutes until the crystals were dissolved. Viable cells were detected by measuring absorbance at 570 nm using MRX II absorbance reader (DYNEX Technologies, Chantilly, Virginia, USA). The cell growth was expressed as a percentage of absorbance in cells with PTL treatment to that in cells without PTL treatment (100%). The inhibition rate (IR) was calculated as follows: IR = (1-A value of PTL well/A value of control well) × 100%

### Flow Cytometry

1 × 10^5 ^cells suspended in 2 ml fresh media were plated in each well of a 6-well flat-bottomed microtiter plate and incubated overnight. Then PTL with indicated concentrations were added. After 48 hours cells were harvested and washed twice with pre-cold PBS and then resuspended in 1× binding buffer at a concentration of 1 × 10^6 ^cells/ml. 100 μl of such solution (1 × 10^5 ^cells) was mixed with 5 μl of annexin V-FITC and 5 μl of Propidium Iodide (PI) (BD Biosciences, San Jose, CA, USA) according to the manufacturer's introduction. The mixed solution was incubated at room temperature (25°C) away from light for 15 minutes. Then 400 μl of 1× dilution buffer was added to each tube. Analysis was performed by Beckman Coulter FC500 Flow Cytometry System with CXP Software (Beckman Coulter, Fullerton, CA, USA) within 1 hour.

### DNA fragmentation analysis

BxPC-3 cells (1 × 10^6 ^cells) were seeded in 6-well microtiter plate. Then the cells were treated with the indicated concentrations of PTL for 48 hours. For analysis of genomic DNA, attached and nonattached cells in the supernatant were harvested and collected together. DNA was extracted by the DNA extraction kit (QIAGEN, German) according to the manufacturer's instruction. 5 μg of DNA was separated on a 2% agarose gel. DNA in the gel was stained with ethidium bromide, visualized under UV light, and photographed.

### Wound closure assay

Cells were plated in 6-well-plates. When the cells grew into full confluency, a wound was created on the monolayer cells by scraping a gap using a micropipette tip and then PTL with indicated concentrations were added immediately after wound creation. The speed of wound closure was compared between PTL treated groups and the control group (PTL untreated cells). Photographs were taken under 100× magnifications using phase-contrast microscopy (OLYMPUS IX70, Olympus, Tokyo, Japan) immediately after wound incision and at later time points as showed.

### Cell invasion assay

A Transwell cell culture chamber (Millipore, Bedford, MA, USA) with a 6.5-mm-diameter polycarbonate filters (8-μm pore size) was coated with Matrigel, dried and reconstituted at 37°C with culture medium. Cells were divided into three groups: the control group, 7.5 μM group and 15 μM PTL group. We placed culture medium containing 20% FBS in the lower chamber (24-well-plates). Then the cells at 1 × 10^5 ^cells per chamber were added to the upper chamber in DMEM containing 10% FBS. After 48 hours incubation at 37°C the suspended media in the lower chamber were removed. The cells that had invaded to the lower side of the filter were fixed in methanol, stained with GIMSA solution. The number of cells that passed through the pores into the lower chamber was counted under a phase-contrast microscope (Leica DMLB2, Leica Microsystems AG, Wetzlar, Germany) (five fields per chamber).

### Western blotting

Proteins were extracted from cultured cells and were subjected to western blot analysis using specific antibodies for bcl-2, caspase-9 and pro-caspase-3 protein. The cells (~2 × 10^8 ^cells) were harvested and rinsed twice with PBS after PTL treatment for 48 hours. Cell extracts were prepared with pre-cold lysis buffer (50 mM Tris-HCl, 150 mM NaCl, 1% Triton X-100, 0.5% deoxycholate, 1 mM EDTA, 1 mM Na3VO4, 1 mM NaF, 2% Cocktail) and cleared by centrifugation at 12000g for 30 minutes at 4°C. Total protein concentration was measured using the BCA assay kit (Sigma) according to the manufacturer's instruction. Cell extracts containing 30 μg of total protein were separated by 12% SDS-polyacrylamide gel electrophoresis (SDS-PAGE), and the proteins were electrotransferred onto nitrocellulose membrane (Millipore, Bedford, MA, USA). The membrane was then blocked with TBST (10 mM Tris-HCl, pH 7.4, 150 mM NaCl, 0.1% Tween-20) containing 5% w/v nonfat milk, and then incubated with primary antibody (dilution factor, 1:1000) in TBST with gentle agitation overnight at 4°C. The membrane was washed 3 times for 10 minutes incubation with TBST and hybridized with redish-peroxidase (HRP)-conjugated secondary antibody (1:2000 dilution, Dakocytomation corporation, Glostrup, Denmark) corresponding to each primary antibody with gentle agitation for 2 hours at room temperature. Protein bands specific for antibody were visualized by enhanced chemiluminescence (Amersham Pharmacia Biotech, Piscataway, NJ, USA).

### Statistical analysis

All the detection items in this study were repeated at least 3 times. Statistical analysis was done using SPSS software (Version 13.0, SPSS Inc, Chicago, IL, USA). The data was expressed as mean ± SD. Statistical significance of the differences between the control- and PTL-treated cells was determined by a two-tailed Student's t test with a 95% confidence interval.

## Results

### PTL inhibited proliferation of the pancreatic cancer cell in a dose-dependent manner

**Figure 1:**
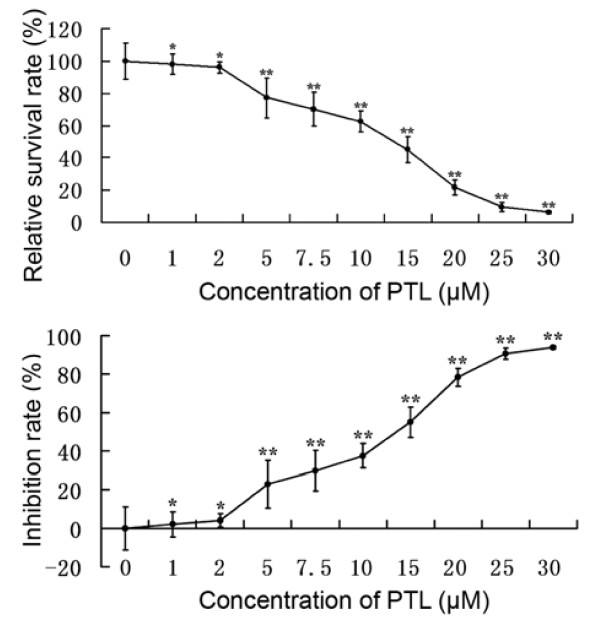
**PTL inhibited BxPC-3 proliferation**. MTT assay demonstrated that PTL can inhibit BxPC-3 cell growth in vitro. Besides, this effect was in a dose-dependent manner. The cell viability and inhibition rates were calculated by comparing with the control group (100%) after 48 hours treatment. Data were presented as mean ± SD (n = 3). Points, mean; bars, + SD. *, P > 0.05; **, P < 0.01 compared with the control group.

The survival and inhibition rate of BxPC-3 cells following treatment with different PTL concentrations was measured. Cells treated with PTL for 48 hours were compared with PTL-untreated cells. The MTT assay (Fig. 1) demonstrated that treatment with 1 μM and 2 μM for 48 hours insignificantly triggered cell death (P > 0.05 VS control). However, concentrations from 5 μM to 30 μM could markedly inhibit tumor cells (P < 0.01 VS control). The bivariate correlation analysis confirmed the negative relationship between PTL concentrations and cell survival rates and the positive relationship between PTL concentrations and cell inhibition rates. In BxPC-3 cells, EC_50 _was estimated to be 14.5 μM.

### PTL induced significant apoptosis in human pancreatic cancer cell

**Figure 2:**
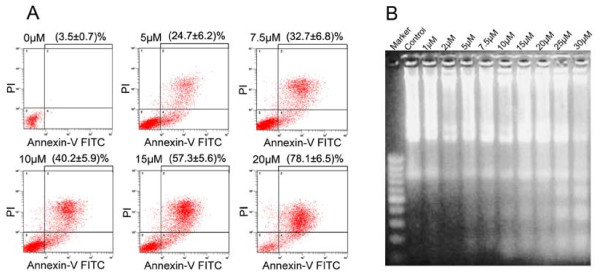
**PTL induced BxPC-3 apoptosis**. BxPC-3 cells were treated with the indicated concentrations of PTL for 48 hours. (A) The quantification of apoptosis was estimated by Annexin-V/PI-FACS analysis. As apoptotic events Annexin-V-positive cells (right quadrant in the density dot plot) were summarized. (B) DNA Fragmentation Analysis indicated that the cells treated with higher concentrations of PTL showed higher proportions of apoptotic internucleosomal DNA fragmentation. These results revealed that PTL-induced apoptosis in BxPC-3 cells was in a dose-dependent manner. The data was described as mean ± SD (n = 3) and the representative figures are shown.

To investigate the effect of inducting apoptosis by PTL in BxPC-3 cells, the flow cytometry and DNA fragmentation analysis were preformed. Annexin-V/PI-FACS analysis (Fig. 2A) was applied to quantify the apoptotic phenotype. Annexin-V-positive cells (right quadrant in the density dot plot) were summarized, including early apoptotic and late apoptotic cell death. PTL-treated cells revealed morphologic events of apoptosis more significantly than cells treated with DMSO alone. The inductive effect of apoptosis presented as a concentration-dependent manner. The apoptosis induced was further confirmed using DNA fragmentation analysis (Fig. 2B). Disintegrated nuclei and nonrandom DNA fragmentation were found on gels. More apoptotic internucleosomal DNA fragmentation was observed after higher concentrations of PTL treatment. These results revealed that PTL effectively induced a dose-dependent apoptosis in human pancreatic cancer cell.

### PTL suppressed BxPC-3 cell migration

**Figure 3:**
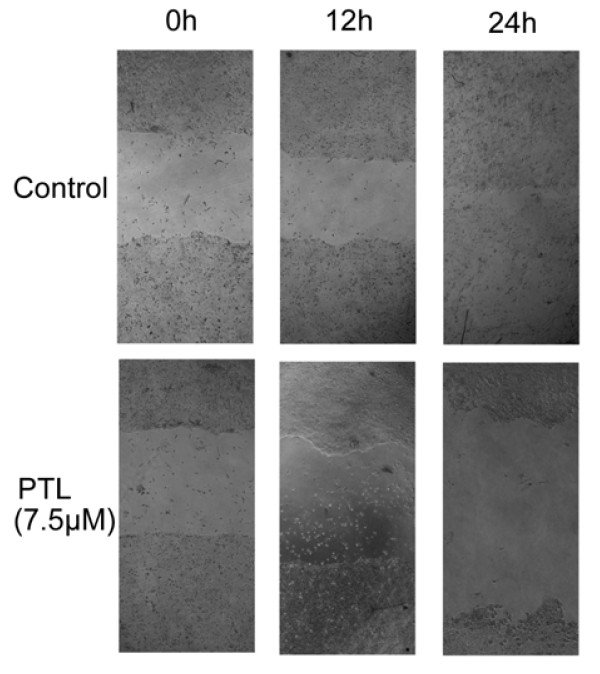
**PTL suppressed BxPC-3 migration**. The wound gap of cells was scratched by a micropipette tip. Cells were incubated in the presence of PTL. 24 hours later the wound gap of BxPC-3 in the control group was nearly closed. On the contrary, after exposure to PTL at 7.5 μM, the speed of wound closure was much slower and the wound was still widely open at twenty-four hours.

Increased migration rate is one of the characteristics in metastatic cancer cells [13]. Pancreatic cancer is a major health problem due to its high risk of metastasis. Accordingly the wound closure assay (Fig. 3) was used to investigate if PTL influenced migration ability of BxPC-3 cells. Wound gap of similar size was created in monolayer BxPC-3 cells at 0 hour. The gap was filled with migrating cells gradually and nearly closed in the control group after 24 hours. Oppositely, the wounds were still widely open at 24 hours after exposure to PTL at indicated concentrations. The results indicated that PTL treatment could inhibit migration of pancreatic cancer cell.

### PTL inhibited BxPC-3 cell invasion

**Figure 4:**
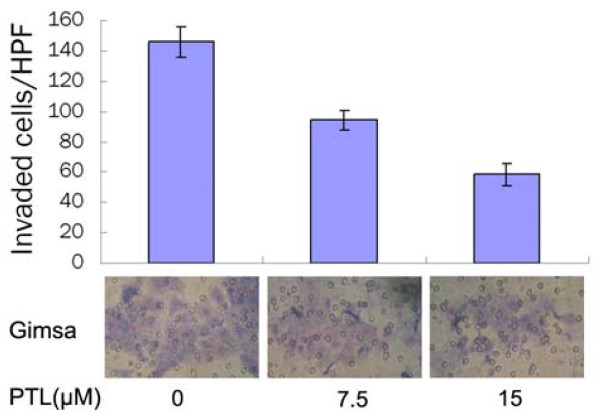
**PTL inhibited BxPC-3 cells invasion**. Cells were fixed, stained and counted at 48 hours. The invasion cell numbers in PTL-treated groups were significantly less than the control group (P < 0.05), which indicated that PTL suppressed cell invasion dose-dependently.

The effect of PTL on BxPC-3 cell invasion was detected by a reconsitituted Matrigel membrane. The number of cells that passed through the filter and into the lower chamber was counted and compared. As a result, PTL at different concentrations obviously inhibited invasive ability of pancreatic cancer cell. The cell numbers of 7.5 μM and 15 μM PTL groups were (94 ± 7)/HPF and (58 ± 8)/HPF respectively, which were less than (146 ± 10)/HPF of control group (P < 0.05) (Fig. 4).

### PTL downregulated Bcl-2 and upregulated Bax expression. No change was found on Bad

**Figure 5:**
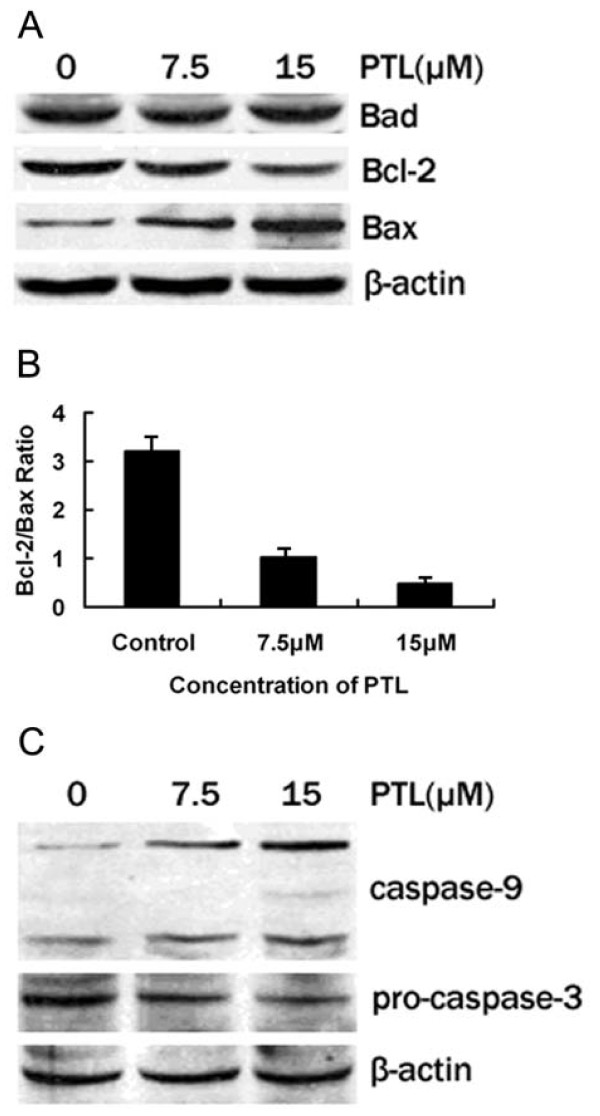
**Apoptosis-related protein expression after PTL treatment for 48 hours**. (A) PTL decreased Bcl-2 expression and induced Bax expression. No obvious change was observed on Bad; (B) Bcl-2/Bax ratio was decreased significantly with the increasing concentration of PTL; (C) Activation of caspase-9 and caspase-3 after PTL treatment.

The underlying mechanism of PTL was also explored in the study. The activation of several apoptosis-related proteins may contribute to PTL-induced apoptosis. In Bcl-2 family members, the expression of Bcl-2, Bax and Bad after PTL treatment for 48 hours were detected by Western blotting (Fig. 5A). PTL obviously decreased the protein expression of antiapoptotic Bcl-2 and increased the protein expression of proapoptotic Bax in the BxPC-3 cells after being treated with indicated concentrations. No change was found on Bad. Therefore, the susceptibility to PTL-induced apoptosis might be attributable to the imbalance of Bcl-2/Bax (Fig. 5B).

### Effect of PTL at various concentrations on caspase-9 and caspase-3 expression

Data have showed many anticancer agents are capable of initiating the caspase activation and inducing apoptosis [14]. Hence the caspase cascade in PTL-induced effect was also analyzed. After PTL treatment at indicated concentrations for 48 hours, Caspase-9 and Pro-caspase-3 expressions of BxPC-3 cell were explored (Fig. 5C). The dose-dependent proteolytic cleavage of caspase-9 was detected. Following caspase-9 activation, Pro-caspase-3 expression was downregulated significantly. Therefore, PTL-induced apoptosis was confirmed to be caspase-dependent.

## Discussion

Pancreatic cancer is a major unsolved health problem because of its biological aggressiveness. In the last decade, traditional clinical cancer therapy regimens as surgical tumor resection, cytotoxic chemotherapy, and radiation therapy have been supplemented with individualized targeted therapies directed against molecular determinants of the tumor. In spite of improved multimodal therapeutic regimens, 5 year survival does not exceed 5 percent. Inherent or acquired resistance towards cytotoxic agents, ionizing radiation, or both, is one of the hallmarks of biological aggressiveness of pancreas cancer as a solid tumor. To develop a new chemotherapeutic agent is still a clinical major concern as well as the better understanding of etiopathogenesis and molecular biology of pancreatic cancer.

NF-kB is ubiquitous and can be detected in the cytoplasm of many cell types. Several researches have indicated that constitutive NF-kB activation may conduce to pancreatic tumorigenesis [15,16]. Hence, the chemotherapeutic potential of NF-kB inhibitors should be evaluated. PTL is one of the traditional medicines extracted from medical herb Feverfew in European and American. Studies have shown that PTL targets NF-kB via inhibition of the upstream regulator IkB kinase (IKK) [17] which phosphorylates IkB and targets it for proteasomal degradation. PTL and its analogues have recently been shown to inhibit proliferation, suppress invasiveness and induce apoptosis of several human cancer cells [4-6,18]. Further studies indicate that in vitro and vivo PTL and its analogues-induced growth inhibition and apoptosis is associated with NF-kB pathway, and the effect is more significant combined with COX inhibitor [12,19]. But the detailed and precise mechanism underlying PTL induced apoptosis remains unclear which attracted our interest.

In our study it was found that PTL significantly inhibited growth of BxPC-3 cells. MTT assay demonstrated a dramatic loss of viability of cancer cell which was treated with PTL in a dose-dependent fashion. Next PTL-induced apoptosis was observed. Flow cytometry indicated that PTL conspicuously induced apoptosis which was confirmed by DNA fragmentation analysis. Meanwhile the migration and invasion assay indicated that PTL effectively suppressed cancer cell movement. Data mentioned above demonstrated PTL might be a novel chemotherapeutic agent.

In order to explore the molecular mechanism of PTL-induced apoptosis in BxPC-3 cell, several genes were detected. Wang et al [20] demonstrated that combination therapy with PTL and arsenic trioxide inhibited the growth of pancreatic cancer cells via the mitochondrial pathway. Researches have reported that Bcl-2 family members are associated with mitochondria-related apoptosis [21,22]. As main members of Bcl-2 family, the antiapoptotic gene Bcl-2 and proapoptotic gene Bax were analyzed in most human cancers which played important roles in tumorigenesis and development [23]. Bcl-2 and Bax localize at the outer membrane of mitochondrial. The balance between them prevents translocation of cytochrome-c from the mitochondria and determines the apoptosis resistance. Inhibition of Bcl-2 or induction of Bax breaks the balance between two genes (as showed in Fig.5B), resulting in mitochondrial dysfunction and cytochrome-c release [21,22]. Researches have demonstrated that several Bcl-2 family members are regulated by NF-kB [24,25]. Promoter analysis showed Bcl-2 had multiple putative NF-kB binding sites [26,27]. Meanwhile, inhibition of NF-kB depressed Bcl-2 expression [28].

Caspases, a family of cysteine proteases, play a critical role in the execution of apoptosis [29] which are modulated by several upstream genes, especially cytochrome-c [30]. Once cytochrome-c is released into cytoplasm, it binds to the adaptor molecule, Apaf-1, and forms the apoptosome that activates caspase-9. Activated caspase-9 cleaves and activates procaspase-3 [31]. In our study data showed that Bcl-2 and procaspase-3 proteins were down-regulated after PTL treatment with the Bax and caspase-9 protein up-regulated. Mitochondrial involvement contributing to the mechanism of PTL-induced apoptosis included NF-kB-mediated Bcl-2 down-regulation and Bax up-regulation, release of mitochondrial cytochrome-c to the cytoplasm and activation of caspase-9 and caspase-3.

In summary, PTL might be a new agent which can effectively inhibit proliferation, invasion and induce apoptosis in pancreatic cancer. Although the molecular mechanisms for the PTL-induced effect still need to be clarified, our data showed that the Bcl-2 family molecules and caspase cascade reaction may be involved. Further studies in vivo should be designed to verify the PTL-induced effect.

## Conclusions

NF-kB inhibitor PTL may be an agent which can effective against pancreatic cancer, because they can effectively inhibit cell proliferation, induce cell apoptosis and suppress metastatic activity. Although the molecular mechanism(s) for the PTL-induced cancer cell apoptosis are poorly understood, the Bcl-2 family molecules and caspase cascade reaction might be involved. Therefore, NF-kB specific inhibitors include PTL may be applicable to a chemotherapeutic strategy for pancreatic cancer. But this possibility should be followed-up with further comprehensive studies.

## Competing interests

The authors declare that they have no competing interests.

## Authors' contributions

JWL, MXC and YX carried out the molecular experiment and drafted the manuscript. QSW and JM carried out the collection of tissue sample. PY and HYX participated in the design of the study and performed the statistical analysis. DSH conceived of the study, and participated in its design and coordination and helped to draft the manuscript. All authors read and approved the final manuscript.
